# Trend of the Burden of Larynx Cancer in Brazil, 1990 to 2019

**DOI:** 10.1590/0037-8682-0269-2021

**Published:** 2022-01-28

**Authors:** Luciana de Paula Viana, Maria Teresa Bustamante-Teixeira, Deborah Carvalho Malta, Gulnar Azevedo e Silva, Meghan Mooney, Mohsen Naghavi, Mário Círio Nogueira, Valéria Maria de Azeredo Passos, Maximiliano Ribeiro Guerra

**Affiliations:** 1 Universidade Federal de Juiz de Fora, Programa de Pós-Graduação em Saúde Coletiva, Juiz de Fora, MG, Brasil.; 2 Universidade Federal de Minas Gerais, Departamento de Enfermagem Materno Infantil e Saúde Pública, Belo Horizonte, MG, Brasil.; 3 Universidade do Estado do Rio de Janeiro, Instituto de Medicina Social, Rio de Janeiro, RJ, Brasil.; 4 University of Washington, Institute for Health Metrics and Evaluation, Seattle, WA, USA.; 5 Faculdade de Ciências Médicas de Minas Gerais, Belo Horizonte, MG, Brasil.

**Keywords:** Larynx câncer, Epidemiology, Mortality, Disability-adjusted life years, Global Burden of Disease

## Abstract

**INTRODUCTION:**

Larynx cancer is one of the most common head and neck cancers, whose main risk factors are smoking and alcohol use, and its occurrence and prognosis depend on adequate and timely preventive measures. This study aimed to investigate the burden of larynx cancer in Brazil and its states.

**METHODS:**

Using estimates from the Global Burden of Disease Study 2019, this study analyzed the trends of incidence, mortality, and disability-adjusted life years (DALYs) for larynx cancer between 1990 and 2019, besides the mortality-to-incidence ratio and the socio demographic index.

**RESULTS:**

Incidence and mortality due to larynx cancer in Brazil, which are approximately eight-fold higher for men, showed a declining trend between 1990 and 2019 (APPC: -0.4% and -1.0%, respectively). The DALYs also showed negative variation between 1990 and 2019 for both sexes in Brazil, mainly due to the decrease in premature deaths, with the greatest reduction in the state of São Paulo. For the states of Brazil in 2019, the higher age-standardized incidence rate (Rio Grande do Sul, 3.83 cases per 100,000 inhabitants) is twice the lowest rate (Piauí, 1.56 cases per 100,000 inhabitants).

**CONCLUSIONS:**

A fall in the burden of larynx cancer was observed in Brazil over the past 30 years, which may be attributed to a reduction in smoking and to an improvement in treatment. However, the regional inequalities in the country remain evident, especially for males. This data can guide public policy priorities to control the disease in Brazil.

## INTRODUCTION

Among the head and neck tumors, larynx cancer ranks third in Brazil and in the world, behind thyroid tumors and the lip and oral cavity tumors^1^ ^-^ ^3^. Around the world, there were 180 thousand new cases in men and 28 thousand in women in 2018, according to estimates from the International Agency for Research on Cancer of the World Health Organization - IARC/WHO^4^. In Brazil, 4,532 deaths by larynx cancer were registered in the Mortality Information System (SIM, in Portuguese) in 2019, which corresponds to nearly 2% of the cancer mortality in the country^5^.

The main risk factors for larynx cancer are smoking and alcohol consumption, especially among the well-differentiated squamous cell carcinomas which represent 98% of the cases^6^ ^,^ ^7^. Some environmental and dietary factors were also associated with an increase in risk for larynx cancer, such as exposure to textile dust, polyaromatic hydrocarbons, asbestos, infection by human papillomavirus (HPV), and consumption of red meat^8^ ^-^ ^11^. In Brazil, there was a high fraction of larynx cancer attributed to selected modifiable risk factors, estimated for the population in 2020, with 80.2% in men and 71.1% in women^8^. 

Symptoms in the initial stage of laryngeal cancer are mild, which makes early diagnosis difficult. However, the disease’s progression can cause sequelae and disabilities, affecting important functions such as swallowing and speech, and resulting in poor survival and quality of life^12^ ^,^ ^13^.

Unfortunately, the majority of the patients are diagnosed in advanced stages of larynx cancer (more than 75% in stage III or IV), especially supraglottic tumors^11^, when the therapeutic options have a notably lower impact on the prognosis^14^ ^,^ ^15^. A 5-year survival rate for patients with early stages of larynx cancer is approximately 80% and 50% for glottic and supraglottic tumors, respectively^11^.

Based on data from the International Agency for Research on Cancer (IARC/WHO), a trend towards a reduction in the incidence (1993-2012) and mortality (1996-2013) of laryngeal cancer in Brazil was found^16^. Although a decreasing trend in mortality was shown in Brazil between 1996 and 2010, based on data from the Mortality Information System, a trend of increased mortality of laryngeal cancer was observed in the North and Northeast regions, for both sexes, which points to regional inequalities^17^. 

The Global Burden of Disease study (GBD) is an international initiative coordinated by the Institute for Health Metrics and Evaluation (IHME), which aims to obtain comparable estimates of the burden of the disease among countries, endeavoring to measure disability and death from a multitude of causes worldwide, thus contributing to evidence-driven health policies at a global level, as well as at regional and national levels^18^ ^,^ ^19^.

This study uses the GBD estimates to evaluate the trend of the burden of larynx cancer in Brazil, its states, and the Federal District between 1990 and 2019 to obtain information most current and at the subnational level that can support the implementation of effective strategies for the disease prevention and control.

## METHODS

This study counted on estimates from the GBD 2019, from the Global Health Data Exchange (GHDx) query tool (http://ghdx. healthdata.org/gbd-results-tool), which includes extensive estimates about 369 diseases from 204 countries and territories, from 1990 to 2019^1^.

To estimate mortality, data from the SIM were used, with adjustments for the under-reporting of deaths and non-specific causes, called garbage codes^20^. To estimate cancer incidence, data from available Brazilian population-based cancer registries (PBCR) were used, and mortality-to-incidence ratio (MIR) for regions without quality or complete PBCR systems^21^. The GBD uses standardized tools to model processed data and generate estimates of each quantity of interest by age, sex, location, and year, such as Cause of Death Ensemble model (CODEm), and a Bayesian meta-regression modelling tool, DisMod-MR 2.1, to ensure consistency between incidence, prevalence, remission, excess mortality, and cause-specific mortality for most causes^1^ ^,^ ^20^.

The GBD uses codes from the International Disease Classification (ICD-10) for definition of larynx cancer: C32-C32.9^21^.

The following estimates per 100,000 in habitants, for Brazil and its states between 1990 and 2019, were considered: age-standardized incidence rate (ASIR), age-standardized mortality rate (ASMR), and disability-adjusted life years (DALYs), a composite indicator that expresses the total burden of diseases by combining in one measure the time lived with disability, Years Lived with Disability (YLD) - prevalence estimates multiplied by disability weights for mutually exclusive sequelae of diseases and injuries, and the time lost due to premature mortality, Years of Life Lost (YLL) - subtracting the age at death from the longest possible life expectancy for a person at that age^1^.

Each estimate was expressed with its 95% uncertainty intervals (95% UIs), which considers errors that may have occurred in the modeling and reflects the uncertainty associated with the size of the samples used as data sources, the adjustments in the data sources for estimate indicators for all causes, as well as uncertainties in estimating model parameters and model specifications for specific causes and all causes^22^. For this, a sample of 1,000 draws was taken from the subsequent distribution of each estimation step and the aggregation of uncertainty across age, sex, and location was done on each draw, assuming independence of uncertainty. The lower and upper uncertainty intervals (UIs) represent the ordinal 25th and 975th draws of each quantity and attempt to describe both the modeling and sampling error^1^ ^,^ ^23^.

To explore regional inequalities, we calculated the MIR for Brazil and its states in the years 1990 and 2019, which was analyzed in relation to the sociodemographic index (SDI) for these regions in the same years. The SDI is a summary measure considered in the GBD study that identifies the level of development of each geographic region, represented by the composite average of the values of per capita income, education level, and fertility rates, expressed by a scale of 0 (lowest level) to 1 (highest level)^24^.

The annual percent changes (APC) and average annual percent changes (AAPC) in incidence and mortality rates in Brazil - with respective 95% confidence intervals (95% CIs) - were calculated, as well as in DALYs and its fractions, using joinpoint regression analysis and the year as the independent variable. Joinpoint regression can show different periods for each estimate by identifying change points in trends. For such analysis, we used the Joinpoint Regression Program software, version 4.8.0.1 - Statistical Research and Applications Branch, National Cancer Institute, Bethesda, USA^25^.

The R program, version 4.1.0 (https://www.r-project.org/) was used to build the maps and the Microsoft Excel program, version 15.0/2013 to the graphics.

The GBD project in Brazil was approved by the Research Ethics Committee from the Federal University of Minas Gerais (UFMG), logged under protocol number 62803316.7.0000.5149.

## RESULTS

An increase was found in the absolute numbers of all laryngeal cancer estimates, for both sexes, in the last 30 years in Brazil. These numbers were 3,326 (95%UI:3,217;3,448) new cases, 2,751 (95%UI:2,652;2,860) deaths and 79,622 (95%UI:76,841;82,613) DALYs in 1990, and increased to 7,681 (95%UI:7,246;8,105) new cases, 5,452 (95%UI:5,136;5,731) deaths and 146,503 (95%UI:138,630;153,963) DALYs in 2019.

**FIGURE 1:**
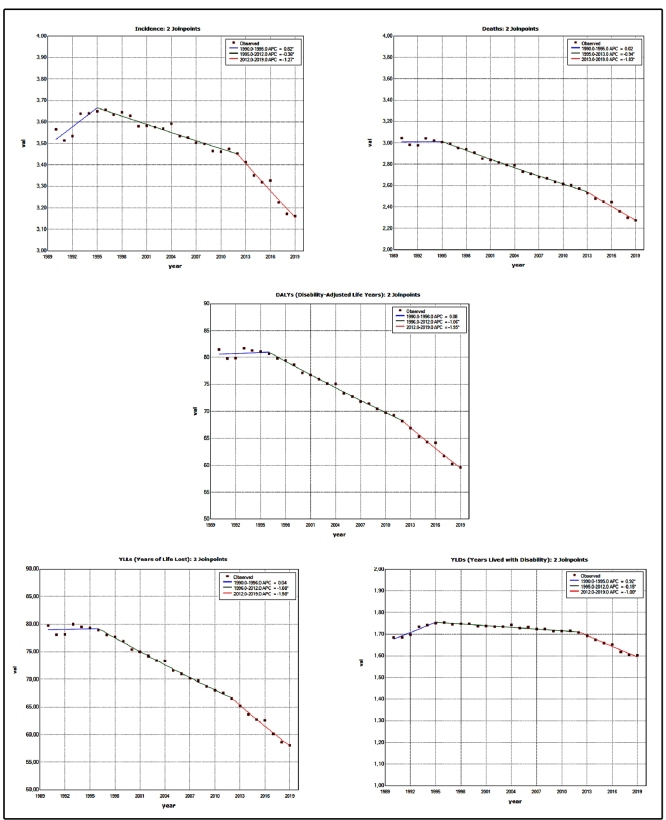
Annual Percentage change (APC) in the incidence, mortality, and DALY(Disability-Adjusted Life Years) rates, and fractions of larynx cancer in both sexes, Brazil, 1990-2019. (*Indicates that the Annual Percent Change (APC) is significantly different from zero at the alpha = 0.05 level. Final Selected Model: 2 Joinpoints).

In Brazil, the ASIR in 1990 and 2019 were respectively 3.57 (95%UI: 3.45;3.70) and 3.16 (95%UI: 2.98;3.33) new cases per 100,000 (Table 1). A trend towards an increase in the incidence rate of larynx cancer from 1990 to 1995 was found, with an APC of 0.8% (95%CI: 0.4;1.3). In the following periods, however, a tendency of decline was identified, with an APC of -0.4% (95%CI: -0.4;-0.3) between 1995 and 2012, and of -1.3% (95%CI: -1.5;-1.0) between 2012 and 2019 (Figure 1). Considering the entire period (1990 to 2019), the incidence of larynx cancer showed a tendency of decline for both sexes, with an AAPC of -0.4% (95%CI: -0.6;-0.2) for women and of -0.3% (95%CI: -0.4;-0.2) for men (Supplementary Material Table 1).

**TABLE 1:** Age-standardized incidence rates (ASIR), age-standardized mortality rates (ASMR) and DALY rates for larynx cancer, according to Brazilian states and regions in 1990 and 2019.

	Incidence(UI 95%)		Mortality (UI 95%)		DALY (UI 95%)	
**REGION**	1990	2019	1990	2019	1990	2019
**Brazil**	3.57	3.16	3.05	2.27	81.44	59.62
	(3.45 - 3.70)	(2.98 - 3.33)	(2.93 - 3.16)	(2.14 - 2.39)	(78.51 - 84.52)	(56.43 - 62.62)
Female	0.88	0.77	0.76	0.56	19.03	13.76
	(0.82 - 0.94)	(0.69 - 0.84)	(0.71 - 0.81)	(0.49 - 0.62)	(17.97 - 20.47)	(12.51 - 15.26)
Male	6.6	6.01	5.67	4.36	150.5	112.98
	(6.36 - 6.85)	(5.63 - 6.39)	(5.44 - 5.90)	(4.07 - 4.60)	(144.98-156.33)	(106.12-119.22)
**NORTH**						
Rondônia	3.76	2.96	3.5	2.28	85.13	57.64
	(3.30 - 4.26)	(2.46 - 3.54)	(3.07 - 3.94)	(1.93 - 2.69)	(73.34 - 96.75)	(48.69 - 68.23)
Acre	1.85	1.95	1.72	1.56	42.89	39.34
	(1.68 - 2.07)	(1.71 - 2.20)	(1.57 - 1.90)	(1.37- 1.76)	(38.65 - 47.80)	(34.59 - 44.32)
Amazonas	3.43	3.75	3.08	2.87	78.56	73.67
	(3.02 - 3.93)	(3.19 - 4.42)	(2.71 - 3.47)	(2.45 - 3.34)	(68.74 - 89.49)	(62.57 - 86.45)
Roraima	3.03	2.43	2.75	1.91	68.4	47.58
	(2.67 - 3.43)	(2.12 - 2.79)	(2.44 - 3.08)	(1.67 - 2.19)	(59.46 - 77.92)	(41.52 - 54.99)
Pará	2.16	1.94	1.96	1.54	49.89	38.69
	(1.85 - 2.51)	(1.66 - 2.25)	(1.68 - 2.28)	(1.32 - 1.80)	(42.55 - 58.59)	(33.20 - 45.37)
Amapá	2.19	2.52	1.89	1.97	47.53	50.52
	(1.98 - 2.39)	(2.23 - 2.83)	(1.72 - 2.07)	(1.74- 2.22)	(43.24 - 52.01)	(44.73 - 57.06)
Tocantins	1.68	2.02	1.52	1.55	36.98	39.33
	(1.40 - 1.96)	(1.66 - 2.45)	(1.28 - 1.78)	(1.29 - 1.84)	(30.80 - 43.95)	(32.55 - 47.04)
**NORTHEAST**						
Maranhão	1.68	1.72	1.57	1.44	43.35	36.9
	(1.34 - 2.09)	(1.39 - 2.12)	(1.22 - 1.96)	(1.16 - 1.76)	(33.61 - 54.87)	(29.46 - 45.38)
Piauí	1.7	1.56	1.51	1.21	37.95	31.12
	(1.47 - 1.96)	(1.34 - 1.82)	(1.29 - 1.72)	(1.04 - 1.41)	(32.70 - 43.53)	(26.66 - 36.45)
Ceará	3.12	3.71	2.72	2.72	70.88	70.12
	(2.60 - 3.81)	(2.90 - 4.61)	(2.23 - 3.31)	(2.19 - 3.33)	(58.34 - 86.69)	(55.91-86.98)
Rio Grande do Norte	2.06	2.63	1.8	1.91	40.15	50.41
	(1.73 - 2.45)	(2.12 - 3.27)	(1.53 - 2.10)	(1.55 - 2.35)	(39.05 - 54.50)	(40.50 - 62.19)
Paraíba	2.69	3.14	2.35	2.3	61.04	61.08
	(2.30 - 3.25)	(2.59 - 3.73)	(2.00 - 2.86)	(1.92 - 2.73)	(51.92 - 74.46)	(50.73-72.92)
Pernambuco	2.43	2.78	2.22	2.18	57.88	57.41
	(2.20 - 2.70)	(2.38 - 3.25)	(2.01 - 2.46)	(1.88 - 2.54)	(52.26 - 64.30)	(49.22 - 67.50)
Alagoas	2.17	2.45	2.02	1.93	52.72	51.65
	(1.91 - 2.60)	(2.11 - 2.87)	(1.78 - 2.39)	(1.64 - 2.25)	(46.33 - 62.65)	(43.63 - 60.45)
Sergipe	2.68	2.85	2.45	2.17	61.87	58.45
	(2.32 - 3.10)	(2.26 - 3.47)	(2.09 - 2.81)	(1.75 - 2.66)	(52.91 - 72.06)	(46.83 - 71.72)
Bahia	2.21	3.03	1.99	2.33	51.75	61.81
	(1.89 - 2.61)	(2.41 - 3.71)	(1.70 - 2.37)	(1.85 - 2.84)	(43.80 - 62.28)	(48.71-75.57)
**SOUTHEAST**						
Minas Gerais	3.0	3.25	2.62	2.3	68.6	61.67
	(2.75 - 3.28)	(2.78 - 3.75)	(2.40 - 2.85)	(1.99 - 2.65)	(62.74 - 74.98)	(53.03 - 71.43)
Espírito Santo	3.15	3.7	2.7	2.63	69.53	71.28
	(2.85 - 3.46)	(3.09 - 4.33)	(2.46 - 2.93)	(2.22 - 3.10)	(63.20 - 76.23)	(59.40 - 84.46)
Rio de Janeiro	4.0	3.04	3.46	2.25	93.61	57.79
	(3.80 - 4.20)	(2.68 - 3.49)	(3.29 - 3.63)	(1.99 - 2.56)	(88.98 - 98.32)	(50.74 - 65.68)
São Paulo	4.8	3.47	4.0	2.37	108.47	62.46
	(4.43 - 5.15)	(2.98 - 3.99)	(3.73 - 4.27)	(2.06 - 2.71)	(101.06-116.29)	(54.07 - 71.52)
**SOUTH**						
Paraná	3.87	3.54	3.37	2.59	87.25	67.08
	(3.63 - 4.13)	(3.02 - 4.09)	(3.16 - 3.60)	(2.20 - 2.99)	(81.95 - 93.58)	(57.01 - 78.01)
Santa Catarina	4.29	3.52	3.59	2.37	93.37	61.35
	(3.92 - 4.70)	(3.01 - 4.11)	(3.30 - 3.92)	(2.05 - 2.76)	(85.51 - 102.27)	(52.57 - 72.30)
Rio Grande do Sul	4.99	3.83	4.05	2.6	110.35	68.95
	(4.69 - 5.31)	(3.30 - 4.45)	(3.81 - 4.29)	(2.26 - 2.97)	(103.92-117.46)	(59.78 - 79.58)
**CENTRAL-WEST**						
Mato Grosso do Sul	3.21	3	2.8	2.29	72.14	59.61
	(2.88 - 3.58)	(2.58 - 3.51)	(2.53 - 3.12)	(1.94 - 2.67)	(64.75 - 80.77)	(50.05 - 69.91)
Mato Grosso	3.05	3.09	2.71	2.32	69.28	59.99
	(2.59 - 3.55)	(2.65 - 3.56)	(2.30 - 3.18)	(1.99 - 2.69)	(58.40 - 82.08)	(51.23 - 69.85)
Goiás	3.27	2.65	2.82	1.93	74.64	50.09
	(2.75 - 3.87)	(2.13 - 3.27)	(2.42 - 3.34)	(1.57 - 2.36)	(62.99 - 89.14)	(40.56 - 62.13)
Distrito Federal	3.44	2.76	2.9	1.82	72.58	43.43
	(2.99 - 3.92)	(2.34 - 3.24)	(2.56 - 3.29)	(1.56 - 2.11)	(63.11 - 83.18)	(36.97 - 51.02)

Rates per 100.000 inhabitants. 95% UI - 95% uncertainty interval.

The ASMRs in 1990 and 2019 were, respectively, 3.05 (95%UI: 2.93;3.16) and 2.27 (95%UI: 2.14;2.39) deaths per 100,000 (Table 1). In the trend analysis, a period of stability in larynx cancer mortality was observed between 1990 and 1995 with an APC: 0.0% (95%CI: -0.4;0.4). In the subsequent periods, a tendency of decline was identified, with an APC of -0.9% (95%CI: -1.0;-0.9) between 1995 and 2013 and of -1.8% (95%CI: -2.1;-1.5) between 2013 and 2019 (Figure 1). Considering the entire period (1990 to 2019), larynx cancer mortality showed a tendency of decline for both sexes, with an AAPC of -0.1% (95%CI: -1.1;-0.9), being -1.0% (95%CI: -1.2;-0.8) for women and -1.1% (95%CI: -1.2;-0.9) for men (Supplementary Material Table 1).

A reduction was identified in the age-standardized rate of DALYs from 81.44 (95%UI: 78.51;84.52) years in 1990 to 59.62 (95%UI: 56.43;62.62) years in 2019. In the trend analysis, a period of stability in the DALYs was observed between 1990 and 1996 with an APC of 0.1% (95%CI: -0.2;0.4). In the periods that followed, there was a tendency of decline, with an APC of -1.1% (95%CI: -1.1;-1.0) between 1996 and 2012 and of -1.9% (95%CI: -2.2;-1.7) between 2012 and 2019 (Figure 1). Considering the entire period (1990 to 2019), the larynx cancer DALYs showed a tendency of decline for both sexes, with an AAPC of -1.0% (95%CI: -1.1;-1.0), being -1.1% (95%CI: -1.2;-0.9) for women and -1.0% (95%CI: -1.1;-0.8) for men, which occurred primarily due to the reduction observed in the YLL fraction during the studied period, with a reduction in potential YLLs due to premature death (Figure 1). The trend analysis of the YLL fraction revealed a period of stability between 1990 and 1996, with an APC of 0.0% (95%CI: -0.3;0.4). In the following periods, there was a tendency of decline, with an APC of -1.1% (95%CI: -1.2;-1.0) between 1996 and 2012 and of -2.0% (95%CI: -2.2;-1.7) between 2012 and 2019. Considering the entire period (1990 to 2019), the larynx cancer YLL showed a tendency of decline for both sexes, with an AAPC of -1.1% (95%CI: -1.2;-1.0), being -1.1% (95%CI: -1.3;-0.9) for women and -1.0% (95%CI: -1.1;0.0) for men. The YLD showed a tendency of increase between 1990 and 1995, with an APC of 0.9% (95%CI: -0.7;1.2). In the subsequent periods, there was a tendency of decline, with an APC of -0.2% (95%CI: -0.2;-0.1) between 1995 and 2012 and of -1.0% (95%CI: -1.1;-0.9) between 2012 and 2019 (Figure 1). Considering the entire period (1990 to 2019), the larynx cancer YLD showed a tendency of decline for both sexes, with an AAPC of -0.2% (95%CI: -0.2;-0.1), being -0.2% (95%CI: -0.3;-0.1) for women and -0.1% (95%CI: -0.2;0.0) for men (Supplementary Material Table 1).

All of the estimates for larynx cancer showed substantially higher values for men. Table 1 shows that men have ASIR and ASMR of approximately eight-fold higher than women with ASIR of 6.01 (95%UI: 5.63;6.39) vs. 0.77 (95%UI: 0.69;0.84) per 100,000 and ASMR of 4.36 (95%UI: 4.07;4.60) vs. 0.56 (95%UI: 0.49;0.62) per 100,000. There was also a predominance of the burden of larynx cancer for males, which shows 8.2-fold more DALYs in males than in females with 112.98 (95%UI: 106.12;119.22) vs. 13.76 (95%UI: 12.51;15.26) per 100,000.

**FIGURE 2:**
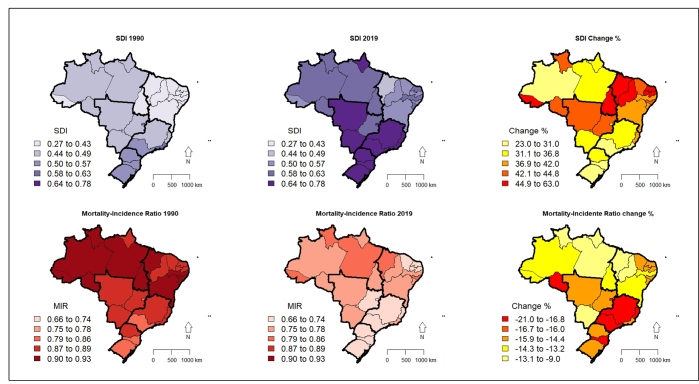
SDI (Socio Demographic Index) and MIR (Mortality-Incidence Ratio) according to Brazilian states and regions in 1990 and 2019, and percentage change (Δ%).

Among the Brazilian states, Amapá showed the highest increases when considering the years 1990 and 2019: from 2.24 (95%UI: 2.03;2.45) to 14.14 (95%UI: 12.49;15.89) for incidence, from 1.84 (95%UI: 1.68;2.01) to 10.50 (95%UI: 9.29;11.85) for mortality, and from 53.02 (95%UI: 47.92;58.5) to 303.39 years (95%UI: 267.16;344.26) for DALYS. Between 1990 and 2019, an increase in SDI and a reduction in MIR were identified in all Brazilian states. In general, the biggest increases in SDI occurred in the states of the North and Northeast regions (with a lower SDI), while the greatest reduction in MIR appeared in the states of the South and southeast regions (with a higher SDI) (Figure 2).

**FIGURE 3:**
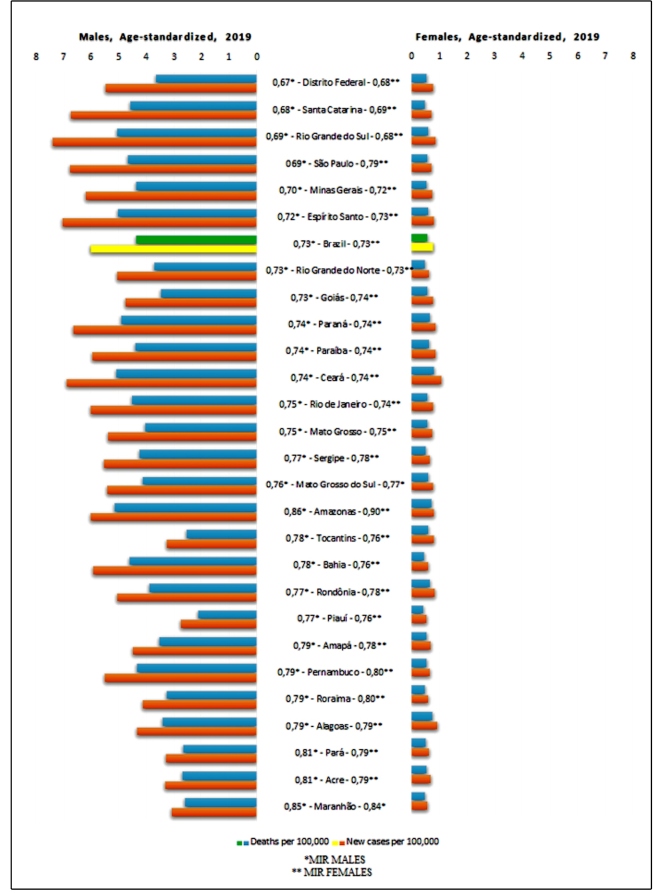
Age-standardized incidence and mortality distributed by sex in the Brazilian states in 2019, in increasing order of MIR (Mortality-Incidence Ratio) in both sexes.

The distribution by sex of the age-standardized incidence and mortality rates for the Brazilian states in 2019 can be seen in Figure 3, in increasing order according to the MIR for both sexes in Brazil in 2019, showing that only the Federal District and five states in the South and Southeast regions (Santa Catarina, Rio Grande do Sul, São Paulo, Minas Gerais and Espirito Santo) had MIR below the national average.

Among the most common types of cancer in Brazil in 2019, the larynx cancer ranked 22nd, reaching 15th in the state of Amazonas. In 2019, the ASIR was higher in the state of Rio Grande do Sul with 3.83 (95%UI: 3.30;4.45) new cases per 100,000, followed by the state of Amazonas with 3.75 (95%UI: 3.19;4.42), Ceará with 3.71 (95%UI: 2.90;4.61), Espírito Santo with 3.70 (95%UI: 3.09;4.33) and Paraná with 3.54 (95%UI: 3.02;4.09) new cases per 100,000. Meanwhile, the lowest rate was found in the state of Piauí with 1.56 (95%UI: 1.34;1.82) new cases per 100,000 (Table 1).

The highest rate of larynx cancer deaths and DALYs in 2019 was verified in the state of Amazonas (2.87 and 73.67 per 100,000, respectively), followed by Ceará (2.72 and 70.12 per 100,000), Espírito Santo (2.63 and 71.28 per 100,000), Rio Grande do Sul (2.60 and 68.95 per 100,000), and Paraná (2.59 and 67.08 per 100,000). Again, the lowest rates were found in the state of Piauí (1.21 and 31.12 per 100,000) (Table 1).

The highest decrease in incidence, death, and DALYs rates of larynx cancer from 1990 to 2019 were found in the state of São Paulo, with an AAPC of -1.1%, -1.8% and -1.9%, respectively. On the other hand, the highest increases in these estimates between 1990 and 2019 were observed in the state of Bahia, with an AAPC of 1.1%, 0.6% and 0.6%, respectively.

**FIGURE 4:**
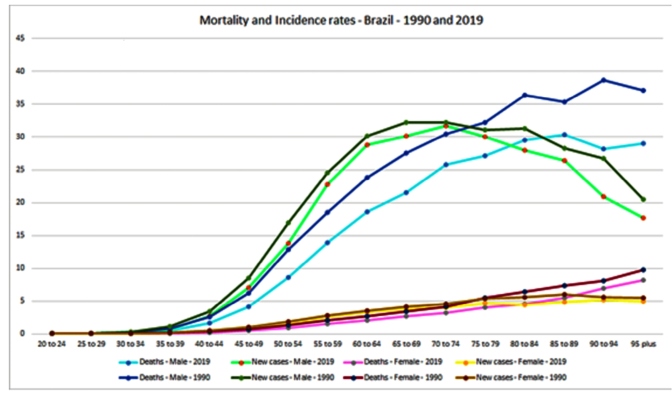
Incidence and mortality rates of larynx cancer by age bracket in Brazil,1990 and 2019.

In terms of distribution by age group, the ASIR for larynx cancer reached its highest value for men between 70 and 74 years of age (31.71, 95%UI:28.79;34.75), and for women between 90 and 94 years of age (5.14, 95%UI:3.55;6.35). The peak of mortality occurred in the age group of 85 to 89 years of age for men (30.36, 95%UI:25.08;34.72), and for women, in the bracket of 95 years of age and over (8.18, 95%UI:5.33;10.21) (Figure 4). Interestingly, we found an increase in the incidence rate between 1990 and 2019 among men aged 20 to 24 years (24.3%, 95%IU:49.0;2.31) **(** Supplementary Material Table 2 **).**

## DISCUSSION

In Brazil, a tendency of decline was verified in ASIR, ASMR, and DALYs for the larynx cancer between 1990 and 2019. These estimates are much higher in men than in women, which is consistent with studies conducted in other countries^26^ ^,^ ^27^, and may be attributable to greater occupational exposure and greater long-term exposure to tobacco in males^12^ ^,^ ^26^ ^,^ ^28^. Moreover, MIR of larynx cancer showed a reduction in Brazil during the studied period (-15%). However, an important geographic variation was observed in the incidence and mortality of larynx cancer.

For a better understanding of this unequal distribution among Brazilian states, changes in MIR rates and SDI were analyzed for each Brazilian state between 1990 and 2019, since MIR has been related to the level of development of a given region^29^. In overall terms, a greater decrease was observed in MIR in the states of regions with the highest SDI, which may suggest that these regions have better conditions to prevent and control the disease. A previous study showed an increase in mortality due to laryngeal cancer, for both sexes, in the North and Northeast regions between 1996 and 2010, although there was a downward trend in mortality in Brazil, emphasizing regional inequalities^17^. This study investigated the trend of larynx cancer mortality in Brazil, with mortality data adjusted for the under-reporting of deaths and non-specific causes (garbage codes), also finding a downward trend, and showing a greater decrease from 2012 onwards.

The disease’s decreasing trend in terms of incidence, mortality, and DALY rates in Brazil throughout the period (also observed in some European countries^26^ and in the world^27^) occurred mainly in the states of the South and Southeast regions of Brazil. A trend towards reduced incidence and mortality for laryngeal cancer in Brazil was also shown in a study that used IARC/WHO data, although in shorter and less current periods (incidence: 1993-2012; mortality: 1996-2013)^16^. 

The reduction in mortality can be explained by improvements in early diagnosis. especially in therapeutic care for larynx cancer, such as more advanced and less invasive surgical procedures, like transoral videolaryngoscopy surgery, combined with more improved radiotherapy and chemotherapy treatments by means of epithelial growth factor receptor inhibitors^30^. Considering that consumption of tobacco is one of the main risk factors for laryngeal cancer^6^ ^,^ ^7^, the reduction in incidence may be related to successful measures developed by the National Tobacco Control Program in Brazil^31^.

Larynx cancer prognosis depends on both precise and timely diagnosis. The survival in patients with advanced larynx cancer is dramatically low and largely attributed to late diagnosis. Symptoms, other than voice hoarseness and alteration in patients with vocal cord involvement, are mostly vague and nonspecific, and can often be underestimated, contributing to the growth of the tumor and the metastases before a final diagnosis can be made. This can inevitably reduce the efficiency of the treatment and results in a more unfavorable prognosis, since larynx cancer can be avoided and cured when identified in its early stages^32^. Late diagnosis must be faced by early detection programs, especially in situations that involve known risk factors^33^.

Rio Grande do Sul is still the Brazilian state with the highest rates of larynx cancer (Table 1), while in Piauí the disease burden remains low. It is worth noting that smoking and daily alcohol consumption are responsible for nearly 90% of larynx cancer mortality^6^, and Rio Grande do Sul stands out in the country in terms of tobacco consumption^28^ ^,^ ^33^. Moreover, the habit of drinking mate tea (or “chimarrão”, as this beverage is known), by hot infusion made with dried and chopped up *Ilex paraguariensis* leaves*,* has also been considered a possible additional cause to explain the higher risk of cancer in the aerodigestive tract in the South region of Brazil. Although thermal injury has been suggested as a responsible factor, the chemical carcinogenesis of mate tea has not been excluded, since it is considered that the herb has an intrinsic carcinogenic and mutagenic component, regardless of the infusion with hot water and the final product^34^.

As far as distribution by age groups is concerned, larynx cancer is a neoplasm whose incidence and mortality rates increase with age^11^, which was also verified in this study. However, we found an increase in incidence rate between 1990 and 2019 among men aged 20 to 24 years. This finding might be related to an increase in alcohol abuse among young people in Brazil in recent years^35^. Studies have also shown an association between laryngeal cancer and HPV in young nonsmokers^36^, although studies in Brazilian populations show a low prevalence of HPV in this topography^37^ ^,^ ^38^.

In addition to causing avoidable morbidity and mortality, larynx cancer imposes a substantial economic burden upon a country, which becomes particularly important in countries like Brazil, where financial resources for health are limited and concentrated in more developed regions. An analysis of the macroeconomic effect of head and neck cancer in such countries as Bangladesh, India, and Pakistan in 2010 estimated that approximately US$17 billion were lost due to these diseases in only one year^39^. A prospective cohort study of adults in eight Southeast Asian countries showed that 30% of the participants had significant financial commitments due to expenses in the treatment of oral and pharynx cancers^40^. A recent retrospective analysis of hospital data revealed that the cumulative cost for the treatment of larynx and oral cavity cancers reached 94.2 million pounds in the United Kingdom, with nearly 95% of those expenses for hospital treatment. Larynx cancer medical care, from diagnosis confirmation to treatment to rehabilitation, demands a multidisciplinary approach and the use of oncological care technology, hospitalization, examinations, highly complex procedures, and human resources that substantially increase costs. Radiotherapy and hospitalization are the main cost factors of larynx cancer, and late diagnosis is one of the factors responsible for this scenario^41^.

Some study’s limitations should be considered such as differences in registries coverage and in data quality between Brazilian regions. Therefore, part of the regional differences seen in laryngeal cancer estimates can be attributed to differences in the coverage of registries and data quality between Brazilian states, although there has been a considerable improvement in these parameters in Brazil in recent decades^5^. Furthermore, regional differences in the distribution of risk factors for laryngeal cancer should also be considered, which was not investigated in this study. 

However important aspects reinforce the evidence obtained in this study, such as the standardization and robustness of data analysis, and the long term of the study period (almost 30 years), which allowed a better characterization of the laryngeal cancer burden in Brazil, and can help guide public policies and assess the effectiveness of prevention and control programs for this disease in Brazil in the last decades. This study also highlights the importance of maintaining and strengthening all measures developed by the National Tobacco Control Program in Brazil as well as urgent regulatory measures aimed at limiting the exposure of the Brazilian population to alcohol, both important potential carcinogens for larynx cancer^31^. 

More studies based on accurate and reliable data, including risk factor analysis, are required to provide a better understanding of the distribution and quality of government policies aimed at preventing and controlling laryngeal cancer in each Brazilian location.
