# Supplementary material for: Trend of the Burden of Larynx Cancer in Brazil, 1990 to 2019
Source: Rev Soc Bras Med Trop. 2022 Jan 28;55(Suppl 1):e0269-2021. doi: 10.1590/0037-8682-0269-2021 (PMC9009424; doi:10.1590/0037-8682-0269-2021)
Supplement: Supplementary file 1 [file 1678-9849-rsbmt-55-s01-e0269-2021-supp1.pdf]

SUPPLEMENTARY TABLE 1: Larynx Cancer, estimates of the percent variations of the Incidence, Mortality, DALY, YLL, and YLD rates per 100,000 inhabitants: Joinpoint regression, Brazil, 1990 – 2019.

		**INCIDENCE**	
		**APC**	**95%CI**
1990 - 1995		0.8*	0.4; 1.3
1995 - 2012		-0.4*	-0.4;-0.3
2012 - 2019		-1.3*	-1.5;-1.0
		**AAPC**	**95%CI**
1990 - 2019	Both sexes	-0.4*	-0.5; -0.3
	Female	-0,4*	-0,6; -0,2
	Male	-0,3*	-0,4; -0,2
		**MORTALITY**	
		**APC**	**95%CI**
1990 - 1995		0,0	-0.4; 0.4
1995 - 2012		-0.9*	-1,0; -0.9
2012 - 2019		-1.8*	-2.1; -1.5
		**AAPC**	**95%CI**
1990 - 2019	Both sexes	-1.0*	-1.1; -0.9
	Female	-1,0*	-1,2; -0,8
	Male	-1,1*	-1,2; -0,9
		**DALY**	
		**APC**	**95%CI**
1990 - 1995		0.1	-0.2; 0.4
1995 - 2012		-1.1*	-1.1; -1.0
2012 - 2019		-1.9*	-2.2; -1.7
		**AAPC**	**95%CI**
1990 - 2019	Both sexes	-1.0*	-1.1; -1.0
	Female	-1,1*	-1,2; -0,9
	Male	-1,0*	-1,1; -0,8
		**YLL**	
		**APC**	**95%CI**
1990 - 1995		0.0	-0.3; 0.4
1995 - 2012		-1.1*	-1.2; -1.0
2012 - 2019		-2.0*	-2.2; -1.7
		**AAPC**	**95%CI**
1990 - 2019	Both sexes	-1.1*	-1.2; -1.0
	Female	-1,1*	-1,3; -0,9
	Male	-1,0*	-1,1; -0,0
		**YLD**	
		**APC**	**95%CI**
1990 - 1995		0.9	-0.7; 1.2
1995 - 2012		-0.2*	-0.2; -0.1
2012 - 2019		-1.0*	-1.1; -0.9
		**AAPC**	**95%CI**
1990 - 2019	Both sexes	-0.2*	-0.2; -0.1
	Female	-0,2*	-0,3; -0,1
	Male	-0,1*	-0,2; -0,0

AAPC: average annual percent change; APC: annual percent change. Final Selected Model: 2 Joinpoints
95% CI: 95% confidence interval
*Statistically significant value in relation to the hypothesis of the absence of a temporal trend for each segment (p<0.0001)
