# Supplementary material for: Trend of the Burden of Larynx Cancer in Brazil, 1990 to 2019
Source: Rev Soc Bras Med Trop. 2022 Jan 28;55(Suppl 1):e0269-2021. doi: 10.1590/0037-8682-0269-2021 (PMC9009424; doi:10.1590/0037-8682-0269-2021)
Supplement: Supplementary file 2 [file 1678-9849-rsbmt-55-s01-e0269-2021-supp2.pdf]

SUPPLEMENTARY TABLE 2: Age-standardized incidence rates (ASIR) for larynx cancer in Brazil, 1990 and 2019, and percentage change between 1990 and 2019 (Δ %), with respective 95% uncertainty intervals, by sex and age group.

Age Group (years)	Sex	ASIR 1990	95% UI	ASIR 2019	95% UI	Δ % 1990-2019	95% UI
**20-24**	Male	0.070	0.061;0.080	0.087	0.077;0.098	24.3	2.3;48.9
	Female	0.039	0.033;0.045	0.038	0.032;0.045	-1.2	-20.8;24.4
	Both sexes	0.054	0.049;0.060	0.063	0.057;0.069	15.8	00.0;31.3
**25-29**	Male	0.109	0.096;0.125	0.110	0.097;0.126	0.6	-17.0;22.5
	Female	0.060	0.053;0.070	0.053	0.046;0.064	-12.3	-27.8;8.9
	Both sexes	0.084	0.076;0.094	0.081	0.073;0.090	-3.7	-16.6;11.6
**30-34**	Male	0.296	0.263;0.340	0.221	0.195;0.251	-25.4	-37.1;-11.1
	Female	0.076	0.065;0.091	0.063	0.052;0.077	-17.5	-34.7;8.8
	Both sexes	0.184	0.167;0.205	0.140	0.127;0.156	-23.6	-33.7;-11.4
**35-39**	Male	1.061	0.944;1.198	0.736	0.639;0.864	-30.6	-42.4;-15.2
	Female	0.204	0.1730.242	0.156	0.130;0.191	-23.6	-38.4;-2.8
	Both sexes	0.622	0.560;0.691	0.439	0.387;0.502	-29.4	-39.5;-17.1
**40-44**	Male	3.337	2.977;3.739	2.684	2.315;3.124	-19.6	-33.9;-4.0
	Female	0.490	0.423;0.563	0.332	0.281;0.402	-32.3	-44.6;-16.7
	Both sexes	1.893	1.713;2.095	1.475	1.283;1.691	-22.1	-34.4;-9.0
**45-49**	Male	8.532	7.753;9.358	7.078	6.279;7.972	-7.0	-29.1;-2.5
	Female	0.993	0.885;1.127	0.763	0.667;0.906	-23.2	-35.2;-6.2
	Both sexes	4.702	4.315;5.116	3.819	3.431;4.275	-18.8	-29.6;-6.1
**50-54**	Male	16.939	15.760;18.295	13.761	12.376;15.388	-18.8	-29.0;-8.1
	Female	1.833	1.606;2.093	1.484	1.289;1.722	-19.0	-32.1;-1.9
	Both sexes	9.197	8.617;9.879	7.361	6.674;8.142	-20.0	-29.3;-9.9
**55-59**	Male	24.568	22.871;26.321	22.733	20.654;25.186	-7.5	-17.7;3.8
	Female	2.781	2.480;3.179	2.571	2.243;2.941	-7.6	-23.2;10.4
	Both sexes	13.211	12.390;14.133	12.072	11.116;13.215	-8.6	-17.7;1.5
**60-64**	Male	30.154	28.247;32.394	28.816	26.381;31.861	-4.4	-14.7;6.5
	Female	3.455	3.116;3.888	3.298	2.841;3.788	-4.5	-22.3;14.1
	Both sexes	16.129	15.154;17.223	15.117	13.904;16.499	-6.3	-15.8;3.7
**65-69**	Male	32.260	30.01734.883	30.176	27.580;33.187	-6.5	-16.9;5.0
	Female	4.092	3.626;4.616	3.790	3.229;4.404	-7.4	-23.1;10.3
	Both sexes	17.408	16.334;18.664	15.848	14.598;17.193	-9.0	-18.3;1.4
**70-74**	Male	32.226	29.951;34.680	31.714	28.788;34.755	-1.6	-12.4;10.7
	Female	4.488	3.982;5.094	4.021	3.432;4.650	-10.4	-26.9;7.2
	Both sexes	17.328	16.211;18.598	16.439	15.138;17.843	-5.1	-14.5;5.5
**75-79**	Male	31.112	28.824;33.655	30.028	27.055;33.057	-3.5	-13.9;8.0
	Female	5.332	4.687;5.964	4.596	3.893;5.240	-13.8	-27.7;1.5
	Both sexes	16.891	15.724;18.135	15.564	14.039;16.941	-7.9	-16.6;1.8
**80-84**	Male	31.342	27.975;34.076	27.987	24.184;31.251	-10.7	-21.4;1.9
	Female	5.597	4.654;6.331	4.401	3.473;5.159	-21.4	-33.0;-6.2
	Both sexes	16.527	14.787;17.893	13.930	12.111;15.447	-15.7	-24.7;-5.7
**85-89**	Male	28.301	24.065;31.625	26.448	21.901;30.132	-6.5	-18.3;7.5
	Female	5.989	4.893;6.913	4.832	3.737;5.738	-19.3	-32.0;-4.1
	Both sexes	14.827	12.665;16.416	12.851	10.668;14.581	-13.3	-22.5;-2.3
**90-94**	Male	26.699	21.996;30.635	20.848	16.124;24.349	-21.9	-33.5;-9.4
	Female	5.620	4.318;6.768	5.137	3.551;6.349	-8.6	-28.7;14.4
	Both sexes	13.376	11.020;15.213	10.615	8.119;12.283	-20.6	-30.7;-9.8
**>95**	Male	20.466	15.392;24.462	17.614	12.638;21.022	-13.9	-26.5;0.6
	Female	5.503	3.854;7.182	5.005	3.226;6.310	-9.1	-32.2;15.9
	Both sexes	10.396	7.741;12.312	9.330	6.637;11.037	-10.3	-23.7;2.0

Rates per 100.000 inhabitants.
95% UI: 95% uncertainty interval.
